# Factors Affecting Adoption of Improved Sweet Potatoes Varieties in Developing Countries: Literature Review

**DOI:** 10.1002/pei3.70108

**Published:** 2026-01-04

**Authors:** Hercidio Tandane, Betty Waized, Florens Turuka

**Affiliations:** ^1^ Department of Agricultural Economics and Agribusiness, College of Economics and Business Studies Sokoine University of Agriculture Morogoro Tanzania; ^2^ Agriculture Research Institute of Mozambique Gaza Mozambique

**Keywords:** developing countries, institutional barriers, socio‐economic factors, sweet potato adoption

## Abstract

This literature review examines the factors influencing the adoption of improved sweet potato varieties (ISPVs) in developing countries. Drawing on 17 studies conducted primarily in Africa and South Asia, the review categorizes influencing factors into socio‐economic, institutional, agronomic, post‐harvest, psychological, geographic, environmental, and consumption‐related domains. It highlights critical variables, including age, farm size, extension services, group membership, and agronomic traits such as yield and drought tolerance. The findings show that most studies rely on quantitative methods and cross‐sectional designs, often neglecting temporal changes in farmers' behaviors and risk aversion. Despite the positive influence of training, education, and extension services, adoption barriers persist due to market instability and regional disparities. Notably, the review highlights the lack of integration between qualitative and quantitative methodologies, as well as the absence of longitudinal studies to explore dynamic adoption patterns. Researchers are advised to explore mixed methods approaches and consider longitudinal analysis and risk aversion factors to better understand the evolving determinants of ISPVs adoption.

## 1 Introduction

Sweet potato ( *Ipomoea batatas* ) is a versatile crop grown for both food and feed in several countries. Ranking seventh in world food production, it follows wheat ( *Triticum aestivum* ), maize ( *Zea mays* ), rice ( *Oryza sativa* ), barley ( *Hordeum vulgare* ), potato ( *Solanum tuberosum* ), and cassava ( *Manihot esculenta* ) (Fongod et al. 2012). This crop plays a critical role in the global food system by meeting food requirements, reducing poverty, and enhancing food security (Brandenberger et al. 2014). Notably, sweet potato has a high yield potential and can be harvested multiple times per year, making it a significant source of income and sustenance (Timothy et al. 2017). However, in many developing countries, farmers continue to face significant challenges in adopting improved crop varieties, including weak extension systems, inadequate credit and input markets, insufficient institutional support, and limited access to information (Dessalegn et al. 2022; Kaliba et al. 2018). These challenges hinder agricultural innovation and affect productivity, nutrition, and resilience (Dong 2021). In this context, understanding ISPVs adoption is important. In this review, ISPVs refer specifically to biofortified Orange‐Fleshed Sweet Potato varieties, which are widely promoted for their high provitamin A content and nutritional benefits (Sohindji et al. 2022).

Farmers' choices regarding ISPVs are crucial factors influencing crop productivity. These choices are shaped by various factors, including socioeconomic factors (Agoh 2021; Jogo et al. 2021; Mbanaso et al. 2011), institutional factors (Kiiza et al. 2012; Kolawole et al. 2017; Mudombi 2013), and agronomic and post‐harvest factors (Adekambi, Okello, Abidin, and Carey 2020; Adekambi, Okello, Rajendran, et al. 2020; Jenkins et al. 2018). The inclusion of these categories is grounded in prior literature, which consistently identifies them as central determinants of technology adoption across regions (Ahoudou et al. 2025; Samuel et al. 2024). While several adoption studies on ISPVs exist, a significant weakness is their predominant focus on cross‐sectional analyses. This approach neglects potential changes in farmers' adoption behavior over time, including changes in risk aversion (Wang and Cheng 2020).

Given the scattered nature of these studies, this review aims to compile and synthesize the findings to provide a comprehensive overview of the key factors influencing the adoption of ISPVs across different agro‐ecological zones and socio‐economic environments. Such insights are important for guiding future research and informing agricultural policymaking and planning. Moreover, explaining the theoretical models used in adoption studies is essential, as these frameworks guide variable selection, shape interpretation of farmer behavior, and ensure conceptual coherence across studies (Montes de Oca Munguia et al. 2021; Rosário et al. 2022). By characterizing farmer studies and identifying their underlying explanatory methods, this review outlines future research directions and identifies factors that can reduce barriers and enhance adoption. To achieve these objectives, this review is guided by two research questions: (1) What types of methods and theories were used to evaluate farmers' adoption of ISPVs? (2) What are the determining factors that influence farmers' adoption of ISPVs?

## 2 Methodology

**FIGURE 1:**
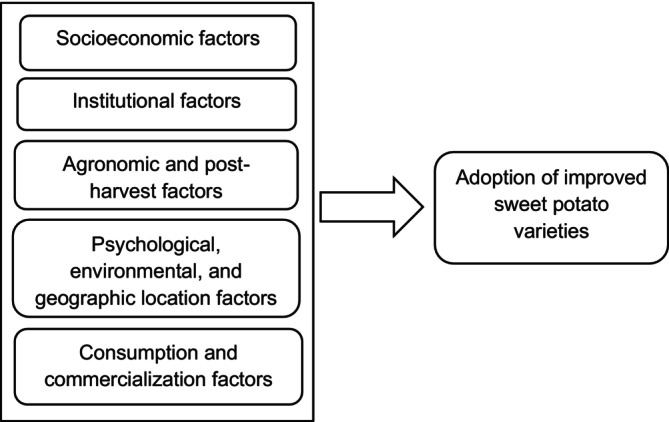
Conceptual framework of factors influencing adoption of ISPVs.

This review presents a narrative review of research literature examining the factors that influence the adoption of ISPVs in developing countries. Relevant literature was predominantly located using Google Scholar searches in 2024 using keywords such as “improved sweet potato varieties” or “orange‐fleshed sweet potato”, “adoption”, “factors” or “determinants”, and “developing countries”. Additional research was identified by analyzing the reference list of selected articles. Studies were included if they examined the determinants of adoption, were conducted in low‐income countries, and aligned with the objectives of the review. Articles written in languages other than English were excluded. Editorials, reviews, posters, book chapters, and publications without full‐text availability were also eliminated. Seventeen articles were selected, covering a variety of countries and approaches. Data from each study were summarized and synthesized thematically into predefined adoption factors (socioeconomic, institutional, agronomic, post‐harvest, psychological, environmental, geographic, consumption, and commercialization) for comparative analysis (Figure 1).

## 3 Study Characteristics

A total of 17 studies on the adoption of ISPVs, published in different journals, were reviewed. Most of the studies were from Africa (16 articles) and South Asia (1 article). In Africa, studies were conducted in the following countries: Mozambique (3 articles), Ghana (3 articles), Nigeria (5 articles), Uganda (2 articles), Kenya (1 article), Zimbabwe (1 article), and Congo (1 article). Moreover, one study was conducted in India. A detailed table summarizing the primary characteristics of the included studies, including the theoretical framework employed and the geographical scope, is provided in Appendix 1 (Table A1).

## 4 Research Designs and Methods Applied in the Reviewed Studies

A total of 16 studies used a cross‐sectional design. Most studies used a quantitative approach to describe factors influencing the adoption of sweet potato varieties and collected data using a structured questionnaire. These studies employed multi‐stage random sampling (12 studies), random sampling (3 studies), and purposive sampling (two studies). Only one study used a qualitative approach, and data were collected through face‐to‐face interviews and focus group discussions.

## 5 Theoretical Models Used in the Studies

Few studies used theoretical models to explain and predict behaviors and outcomes of their studies (7 studies). For example, Mazuze (2007), Adekambi, Okello, Rajendran, et al. (2020), Jogo et al. (2021), and Kiiza et al. (2012) used utility maximization theory to evaluate farmers' decision‐making processes regarding the adoption of agricultural technologies. Jenkins et al. (2018) and Acheampong et al. (2018) used diffusion of innovation theory, which consists of a series of choices and actions over time through which an individual evaluates a new idea and decides whether to incorporate it into an ongoing practice. The focus of this theory is on the adoption process and factors influencing the spread of innovations (Rogers and Williams 1983). A Lancaster's model of consumer choice and the random utility models were used in the Namirimu et al. (2024) study. Lancaster proposed that customers derive satisfaction not only from the good itself but also from its characteristics. Notably, 10 of the reviewed articles did not mention a theoretical model supporting their findings (see Table S1 in the Supporting Information).

## 6 Socioeconomic Factors Influencing Adoption of ISPVs

Fourteen socio‐economic factors were found to be significant in 17 different studies. Table 1 shows that age, education, gender, farm size, household size, marital status, and farming experience are the most studied variables. Likewise, land ownership and the number of children under 5 years are reasonably well‐studied factors. Investigators seem to be less concerned about the influence of off‐farm income, labor availability, occupation, annual income, farm income, area under sweet potato cultivation, and the presence of pregnant or lactating women on the adoption of ISPVs.

**TABLE 1:** Socioeconomic factors studied (*N* = 17).

Variable	Studied	Significant		Effect (sign)
		Positive	Negative	
Age	16	4	2	+/−
Education	16	8	0	+
Gender	14	1	0	+
Marital status	7	0	0	NA
Household size	10	2	0	+
Labor availability	3	1	0	+
Occupation	3	0	1	−
Farming experience	8	3	1	+/−
Annual income	3	2	0	+
Farm income	3	2	0	+
Off‐farm income	4	1	0	+
Livestock ownership	2	1	0	+
Farm size	11	3	0	+
Land ownership	6	2	0	+
Area under sweet potato	3	2	0	+
Number of children/presence of children under 5 years	6	1	0	+
Presence of pregnant/lactating woman	1	0	0	NA

Abbreviation: NA, not applicable.

The positive influence of education has been reported in various studies (Acheampong et al. 2018; Agoh 2021; Jogo et al. 2021; Kolawole et al. 2017; Mbanaso et al. 2011; Namirimu et al. 2024; Okeke et al. 2020; Srinivas and Nedunchezhiyan 2020). The more educated the head of the household is, the more likely they are to understand and adopt new agricultural technologies more quickly than those without education (Djibo and Maman 2019; Mwangi and Kariuki 2015).

Age is one of the human capital characteristics that has been positively associated with the adoption of ISPVs across various studies (Acheampong et al. 2018; Adekambi, Okello, Abidin, and Carey 2020; Adekambi, Okello, Rajendran, et al. 2020; Jogo et al. 2021). It is believed that older farmers have more life experience and expertise, making them more qualified to assess technological information than younger farmers (Ayenew et al. 2020; Ainembabazi and Mugisha 2014). Conversely, some findings confirmed that age negatively influences the adoption of ISPVs (Agoh 2021; Mbanaso et al. 2011). This goes against expectations because sweet potatoes require less labor and resources to produce than other crops, such as cassava, yams, and cocoyam (Mbanaso et al. 2011). Agoh (2021) justifies his findings, which is logical because older adults are usually less active and may find it challenging to participate in activities that demand much energy. Older farmers typically stick to traditional production methods and are generally less open to change (Yokamo 2020; Adeola et al. 2019).

The farm size showed a positive and significant impact in some studies (Ekwe and Onunka 2006; Mbanaso et al. 2011; Namirimu et al. 2024), indicating that farmers holding a large farm size are more likely to adopt ISPVs because they can afford to set aside a portion of their property for experimentation. Similarly, farming experience was found to be positively and significantly correlated with adoption, suggesting that farmers with more experience are better able to overcome challenges and increase their yield through the adoption of improved agricultural technologies (Kiiza et al. 2012; Mbanaso et al. 2011; Okeke et al. 2020). However, this contradicts Acheampong et al. (2018), who found that more experienced farmers are less likely to adopt ISPVs.

Few studies on household size reported a positive impact on adoption (Agoh 2021; Mbanaso et al. 2011), indicating that farmers with larger families are more likely to adopt new agricultural technologies. Likewise, annual and farm income were found to be positively associated in some studies (Agoh 2021; Ekwe and Onunka 2006; Kiiza et al. 2012; Okeke et al. 2020). The positive influence of land ownership has been mentioned in two studies (Jogo et al. 2021; Kolawole et al. 2017). This implies that a stable and reliable tenure system, characterized by inheritance and purchase, will enhance the adoption of agricultural technologies. This is because these tenure systems ensure permanent and secure residency for respondents (Kolawole et al. 2017).

The area under sweet potato cultivation has a positive association with the adoption of sweet potato varieties. Mazuze (2007) found that farmers who cultivated a larger area of sweet potatoes were more likely to adopt improved sweet potato technologies. Similar to the results from this study, Srinivas and Nedunchezhiyan (2020) identified the area under sweet potato cultivation as a variable influencing the adoption of ISPVs in India.

The role of labor availability, off‐farm income, livestock ownership, and the presence of children under 5 years on adoption seems positive but not enough to explain, as only a small number of studies have included these factors. One assumption of adoption studies is that variables related to the adoption of agricultural technologies should not be generalized (Schulz and Börner 2023). This means that the influence of independent variables and their interactions on farmers' technology adoption varies across areas (Xie and Huang 2021). Consistent with this point, Agoh (2021) found a significant adverse effect of age on the adoption of sweet potato technologies in Imo State, Nigeria. However, Okeke et al. (2020) observed a positive and significant association between age and the adoption of improved sweet potato production technologies in the South East of Nigeria.

## 7 Institutional Factors Influencing Adoption of ISPVs

Thirteen institutional variables were found to be significant in 17 different studies. As shown in Table 2, the most frequently examined institutional factors included access to extension services, group membership, training access, and access to credit.

**TABLE 2:** Institutional factors studied (*N* = 17).

Variable	Studied	Significant		Effect (sign)
		Positive	Negative	
Group membership	9	4	0	+
Extension service	10	6	0	+
Training access	7	6	0	+
Access to credit	3	1	0	+
Access of information	2	1	0	+
Marketing distance	2	0	0	NA
Distance to the nearest agricultural field office	2	0	1	−
Distance to the nearest main road	2	0	0	NA
Field to house distance	1	0	0	NA
Holds a leadership position	2	0	0	NA
Access to planting material	1	1	0	+
Frequency of vines distribution	1	0	0	NA
Participation in cooking demonstrations	1	1	0	+
Participation in program/project activities	2	1	0	+
Participation in on‐farm trials	1	1	0	+
Irrigation use	1	1	0	+
Input exchange	1	1	0	+
Vine purchase	1	1	0	+

Abbreviation: NA, not applicable.

Multiple studies found a positive influence of extension services on ISPVs (Adekambi, Okello, Abidin, and Carey 2020; Agoh 2021; Kiiza et al. 2012; Kolawole et al. 2017; Mudombi 2013; Okeke et al. 2020). Regular contact between farmers and extension agents increases farmers' awareness of improved agricultural technologies, thereby enhancing adoption (Norton and Alwang 2020; Wang et al. 2020; Cafer and Rikoon 2018). This aligns with Okeke et al. (2020), who noted that frequent contact with extension agents increases farmers' awareness of new technologies and their use to improve their livelihoods.

Group membership and access to training have generally had a positive influence in several studies (Adekambi, Okello, Abidin, and Carey 2020; Adekambi, Okello, Rajendran, et al. 2020; Agoh 2021; Kiiza et al. 2012; Kolawole et al. 2017; Okeke et al. 2020). These results suggest that belonging to certain groups significantly increases the likelihood of adopting new agricultural technologies, as access to improved varieties is facilitated by farmers' networks, such as farmers' groups (Muange et al. 2014; Tadesse et al. 2017). Mudombi (2013) observed that farmers who participated in training programs were more likely to adopt ISPVs and to have a positive attitude toward them. Likewise, Kiiza et al. (2012) found a positive influence on farmers who participated in plant breeding and varietal selection.

The variable “access to credit” has a positive and significant effect on the adoption of sweet potato technologies. This implies that access to credit enables farmers to buy the inputs required for these new technologies. Agoh (2021) showed that farmers with better access to credit are more likely to adopt sweet potato value addition. It is commonly believed that the closer a household is to the nearest agricultural field office, the higher the likelihood of adoption (Krishnan and Patnam 2014). This is consistent with Adekambi, Okello, Rajendran, et al. (2020), who used the nearest agricultural field office as a proxy variable for access to extension advice and found a negative influence.

Several studies (Adekambi, Okello, Rajendran, et al. 2020; Jenkins et al. 2018; Jogo et al. 2021; Kaguongo et al. 2012; Mudombi 2013) reported that access to planting material, participation in cooking demonstrations, participation in program or project activities, participation in on‐farm trials, irrigation use, input exchange, and vine purchase have a positive and significant impact on adoption. The positive correlation between irrigation use and ISPVs adoption can be explained by the technology's inherent characteristics (Mudombi 2013). Additionally, he observed that farmers involved in project activities are more likely to have a positive attitude toward the new agricultural technology. Adekambi, Okello, Rajendran, et al. (2020) detail the extensive efforts undertaken by the project to educate farmers in Ghana and Nigeria about the health benefits of consuming ISPVs. These efforts included various strategies such as raising market awareness, broadcasting radio programs, creating cookbooks, conducting cooking demonstrations, and training trainers to promote the adoption of ISPVs.

## 8 Agronomic and Post‐Harvest Factors Influencing Adoption of ISPVs

Eleven agronomic and post‐harvest factors were found to be significant in 17 different studies. Table 3 shows that maturity, yield, drought tolerance, pest resistance, and disease resistance are the most studied variables. Furthermore, dry matter, quantity produced, sweet potato output, and sweet potato storage are relatively well‐studied factors. Researchers seem not to be interested in other variables, as they were mentioned in only seven studies, most of which were conducted in the last six years (Adekambi, Okello, Rajendran, et al. 2020; Jenkins et al. 2018; Mazuze 2007; Mudombi 2013; Mugumaarhahama et al. 2021; Namirimu et al. 2024; Srinivas and Nedunchezhiyan 2020).

**TABLE 3:** Agronomic and post‐harvest factors studied (*N* = 17).

Variable	Studied	Significant		Effect (sign)
		Positive	Negative	
Dry matter	2	1	0	+
Maturity	5	5	0	+
Yield	5	4	0	+
Drought tolerance	4	3	0	+
Pest and disease resistance	5	2	0	+
Quantity produced	2	1	0	+
Experience with varieties	1	0	0	NA
Easy to establish with scarce rain	1	1	0	+
Easy to conserve vines during the long dry period	1	0	0	NA
Variety growing	1	0	1	−
Production method	1	0	1	−
Crop establishment	1	0	0	NA
Sweet potato storage	2	1	1	+/−
Storage performance	1	1	0	+
Easy to store in the ground	1	0	1	−
Multiplication and retention capacity	1	1	0	+
Appropriateness to the farming system	1	0	0	NA
Cropping system	1	0	0	NA
Access to planting material	1	0	0	NA
Soil fertility status	1	0	0	NA
Type of production system	1	1	0	+

Abbreviation: NA, not applicable.

Positive influence of maturity and yield on the adoption of new technologies has been reported in several studies (Adekambi, Okello, Abidin, and Carey 2020; Adekambi, Okello, Rajendran, et al. 2020; Jogo et al. 2021; Srinivas and Nedunchezhiyan 2020). Early maturity is important because varieties that mature quickly enable farmers to bridge the hunger gap, the period before other staple crops are ready for harvest (Jogo et al. 2021). These results support those of Adekambi, Okello, Rajendran, et al. (2020), who found that early maturity allows the crop to avoid drought and complete its growing cycle. Similarly, a 10% increase in crop yield results in a 4.8% rise in the geometric mean of the adoption quotient (Srinivas and Nedunchezhiyan 2020).

As expected by Jogo et al. (2021), dry matter content has a positive and significant effect on the adoption of orange‐fleshed sweet potato varieties. Additionally, drought tolerance and pest and disease resistance were found to be positive factors in different studies (Adekambi, Okello, Rajendran, et al. 2020; Jenkins et al. 2018; Jogo et al. 2021; Mudombi 2013). Agronomic traits are more crucial for the retention of the improved varieties (Jogo et al. 2021; Mbusa et al. 2018). These varieties require less water than traditional varieties (Jenkins et al. 2018; Assefa et al. 2020). This is consistent with Mudombi (2013), who found that improved perceptions regarding planting material's ability to resist drought periods enhance both its use intensity and the likelihood of adoption.

Regarding the sweet potato output, easy to establish with scarce rain, storage performance, multiplication, and retention capacity, the type of production system has a positive impact on the adoption of improved varieties (Adekambi, Okello, Rajendran, et al. 2020; Ekwe and Onunka 2006; Mudombi 2013; Srinivas and Nedunchezhiyan 2020). The coefficients for variety growing, production method, and easy storage in the ground were negative and statistically significant (Adekambi, Okello, Rajendran, et al. 2020; Namirimu et al. 2024). The adverse effect of sweet potato storage implies that farmers who do not store sweet potatoes are more likely to sell most of their produce at harvest, especially those with medium to large plots who primarily grow for the market (Namirimu et al. 2024). Jenkins et al. (2018) found that ISPVs can be conserved on the farm for a shorter period compared to traditional varieties due to their high susceptibility to pests and poor resistance to sunlight.

## 9 Psychological, Environmental, and Geographic Location Factors Influencing Adoption of ISPVs

Although psychological, environmental, and geographic location variables have been identified as essential in certain adoption studies (Table 4), they appear less frequently than the previously discussed variables. A positive effect between knowledge of vitamin A and the adoption of ISPVs was reported by Jogo et al. (2021) and Kaguongo et al. (2012). This suggests that farmers who are aware of vitamin A have considerably increased their adoption of the varieties. Acheampong et al. (2018) and Mazuze (2007) found that awareness positively increased the dissemination of improved sweet potato technologies. Similarly, farmers with knowledge of sweet potato processing were more likely to adopt ISPVs (Agoh 2021).

**TABLE 4:** Psychological, environmental, and Geographic location factors studied (*N* = 17).

Variable	Studied	Significant		Effect (sign)
		Positive	Negative	
Knowledge Vita A	2	2	0	+
Awareness of sweet potato	2	1	0	+
Processing experience	2	1	0	+
Vine constrained	1	0	1	−
Cosmopolitaness	1	0	0	NA
Environmental conditions	1	1	0	+
Average annual precipitation	1	1	0	+
Location of the farmer	1	1	0	+

Abbreviation: NA, not applicable.

The positive influences of environmental conditions, average annual precipitation, and the farmer's location have been reported in various studies (Jenkins et al. 2018; Kaguongo et al. 2012; Mazuze 2007). The positive impact of the regional location of the farmer indicates that a farmer in Busia district is 54 times more likely to adopt ISPVs than a farmer in Rachuonyo district (Kaguongo et al. 2012). This can be attributed to the fact that sweet potato is more commercialized in Rachuonyo district than in Busia district, and yields of local varieties grown in Rachuonyo are lower than those of improved varieties (Kaguongo et al. 2012).

## 10 Consumption and Commercialization Factors Influencing Adoption of ISPVs

A few studies (a total of 8) emphasize the importance of consumption and commercialization variables on the adoption of agricultural technologies (Table 5). Consumption variables such as taste (Adekambi, Okello, Rajendran, et al. 2020; Jogo et al. 2021), ease of cooking (Adekambi, Okello, Rajendran, et al. 2020), organoleptic qualities (Jenkins et al. 2018), as well as commercialization variables such as unstable markets (Jenkins et al. 2018), processing equipment (Agoh 2021), value addition (Kaguongo et al. 2012), and market price (Srinivas and Nedunchezhiyan 2020) have been highlighted. A positive influence of taste was observed in the initial adoption decision, as reported by Jogo et al. (2021). This is consistent with Adekambi, Okello, Rajendran, et al. (2020), who found a powerful effect of taste on the adoption of the Apomuden variety. Surprisingly, this study found an inverse relationship between ease of cooking and the adoption of new varieties. The adverse effect of market prices on adoption suggests that as market prices rise, the adoption of ISPVs decreases.

**TABLE 5:** Consumption and commercialization factors studied (*N* = 17).

Variable	Studied	Significant		Effect (sign)
		Positive	Negative	
Taste	2	1	1	+/−
Very sugary/sweet	2	1	0	+
Ease of cooking	2	0	1	−
Sweet potato root consumption	1	0	0	NA
Organoleptic qualities	1	1	0	+
Output sold	2	0	0	NA
Unstable markets	1	1	0	+
Cost of processing	1	0	0	NA
Processing equipment	1	1	0	+
Value addition	1	1	0	+
Market price/Output price	1	0	1	−

Abbreviation: NA, not applicable.

## 11 Conclusions

This review focuses on research concerning farmers and their interaction with improved sweet potato varieties. The aim is to understand the key factors influencing the adoption of these varieties, to enhance the existing knowledge base, and to encourage further research in this field. Therefore, this literature highlights the complex interplay of factors influencing farmers' adoption of ISPVs, emphasizing the critical roles of socioeconomic, institutional, agronomic, post‐harvest, psychological, environmental, and geographic location factors, as well as consumption and commercialization dynamics. While these categories provide a robust framework for understanding adoption, significant gaps remain in the literature. Notably, the absence of studies incorporating mixed method approaches and the limited exploration of risk aversion factors across multiple seasons constrain our understanding of the dynamic and multifaceted nature of adoption decisions. Furthermore, the study relied exclusively on Google Scholar for data retrieval, which may have restricted the breadth of the literature captured. In addition, the review included only English language articles, thus introducing linguistic bias and potentially excluding relevant studies published in other languages. Chapters, editorials, reviews, posters, and sources without full text were also excluded, potentially further narrowing the scope of the evidence. Also, the focus on a single crop limits the generalizability of insights to broader agricultural innovation contexts. Finally, the research syntax employed may not have captured all relevant studies, as the search terms did not explicitly incorporate broader keywords related to developing countries or improved varieties. This may have reduced the discoverability of pertinent literature.

Future research should prioritize integrating qualitative and quantitative methodologies to achieve a holistic understanding of adoption processes. Additionally, incorporating longitudinal analyses of risk aversion can provide valuable insights into how farmers' perceptions evolve and influence their decision‐making over time. Also, future reviews should adopt more comprehensive and systematic approaches, such as PRISMA guidelines for meta‐analyses, to ensure inclusion of diverse databases, languages, and publication types. More refined and comprehensive search strings should be applied to ensure the retrieval of studies across diverse contexts. Addressing these gaps will not only enhance the theoretical knowledge base but also inform the development of context‐specific interventions and policies. Such efforts are essential for promoting the sustainable adoption of ISPVs, particularly in the face of uncertainties that characterize many agricultural systems. This comprehensive approach will ultimately empower farmers, improve productivity, and contribute to food security in diverse agricultural settings.

## Funding

This study was funded by the Regional Scholarship and Innovation Fund (RSIF) of the Partnership for Skills in Applied Sciences, Engineering and Technology (PASET) (Project Grant No. B8501G30221).

## Conflicts of Interest

The authors declare no conflicts of interest.

## Supporting information


**Table S1:** Factors influencing adoption of ISPVs.

## Data Availability

The data that supports the findings of this study are available in the Supporting Information of this article.

## Appendix 1 Summary of Included Studies

**TABLE A1:** Main characteristics of the included studies.

Author	Commodity	Region	Population studied	Data type	Theory used	Number of observations
Acheampong et al. (2018)	Sweet potato	Ghana	Farmers	Cross‐sectional	Diffusion of innovations theory	526
Adekambi, Okello, Abidin, and Carey (2020)	Sweet potato	Ghana and Nigeria	Smallholder farm households	Cross‐sectional	—	345 and 381
Adekambi, Okello, Rajendran, et al. (2020)	Sweet potato	Ghana	Smallholder farm households	Cross‐sectional	Utility maximization theory	262
Agoh (2021)	Sweet potato	Nigeria	Sweet potato processors	Cross‐sectional	—	96
Ekwe and Onunka (2006)	Sweet potato	Nigeria	Farmers	Cross‐sectional	—	150
Jenkins et al. (2018)	Sweet potato	Mozambique	Farmers	FGDs	Diffusion of innovations theory	95
Jogo et al. (2021)	Sweet potato	Mozambique	Smallholder farmers	Cross‐sectional	General utility maximization framework	1538
Namirimu et al. (2024)	Sweet potato	Uganda	Sweet potato growers	Cross‐sectional	Utility maximization behavior	792
Kaguongo et al. (2012)	Sweet potato	Kenya	Farmers	Cross‐sectional	—	340
Kiiza et al. (2012)	Sweet potato	Uganda	Farmers	Cross‐sectional	Utility maximization theory	180
Kolawole et al. (2017)	Sweet potato	Nigeria	Farmers	Cross‐sectional	—	80
Mbanaso et al. (2011)	Sweet potato	Nigeria	Farmers	Cross‐sectional	—	270
Mazuze (2007)	Sweet potato	Mozambique	Sweet potato growers	Cross‐sectional	Utility maximization theory	150
Mudombi (2013)	Sweet potato	Zimbabwe	Smallholder farmers	Cross‐sectional	—	83
Mugumaarhahama et al. (2021)	Sweet potato	Congo	Smallholder farmers	Cross‐sectional	—	360
Okeke et al. (2020)	Sweet potato	Nigeria	Small‐scale farmers	Cross‐sectional	—	100
Srinivas and Nedunchezhiyan (2020)	Sweet potato	India	Sweet potato farmers	Cross‐sectional	—	330
