# Supplementary material for: Factors Affecting Adoption of Improved Sweet Potatoes Varieties in Developing Countries: Literature Review
Source: Plant Environ Interact. 2026 Jan 4;7(1):e70108. doi: 10.1002/pei3.70108 (PMC12766156; doi:10.1002/pei3.70108)
Supplement: Supplementary file 1 — Table S1: Factors influencing adoption of ISPVs. [file PEI3-7-e70108-s001.docx]

**Table S7: Factors influencing adoption of ISPVs**

| **Key factors** | **Examples** | **Number of studies** | **Effects** |
| --- | --- | --- | --- |
| **Socioeconomic** | **Age** (Acheampong et al., 2018; Adekambi et al., 2020a; Adekambi et al., 2020b; Agoh, 2021; Ekwe, 2006; Jenkins et al., 2018; Jogo et al., 2021; Josephine Namirimu et al., 2024; Kaguongo et al., 2012; Kolawole et al., 2017; Mbanaso et al., 2011; Mazuze, 2007; Mudombi, 2013; Mugumaarhahama et al., 2021; Okeke et al., 2020; Srinivas & Nedunchezhiyan, 2020) | 16 | +/- |
|  | **Education level** (Acheampong et al., 2018; Adekambi et al., 2020a; Adekambi et al., 2020b; Agoh, 2021; Ekwe, 2006; Jenkins et al., 2018; Jogo et al., 2021; Josephine Namirimu et al., 2024; Kaguongo et al., 2012; Kiiza et al., 2012; Kolawole et al., 2017; Mbanaso et al., 2011; Mazuze, 2007; Mugumaarhahama et al., 2021; Okeke et al., 2020; Srinivas & Nedunchezhiyan, 2020) | 16 | + |
|  | **Gender** (Acheampong et al., 2018; Adekambi et al., 2020a; Adekambi et al., 2020b; Ekwe, 2006; Jenkins et al., 2018; Jogo et al., 2021; Josephine Namirimu et al., 2024; Kaguongo et al., 2012; Kolawole et al., 2017; Mbanaso et al., 2011; Mazuze, 2007; Mudombi, 2013; Mugumaarhahama et al., 2021; Okeke et al., 2020) | 14 | + |
|  | **Mariatl status** (Acheampong et al., 2018; Agoh, 2021; Ekwe, 2006; Jenkins et al., 2018; Kolawole et al., 2017; Mbanaso et al., 2011; Okeke et al., 2020) | 7 | NA |
|  | **Households size** (Acheampong et al., 2018; Agoh, 2021; Ekwe, 2006; Josephine Namirimu et al., 2024; Kiiza et al., 2012; Mbanaso et al., 2011; Mazuze, 2007; Mudombi, 2013; Mugumaarhahama et al., 2021; Okeke et al., 2020) | 10 | + |
|  | **Labor availability** (Kaguongo et al., 2012; Mugumaarhahama et al., 2021; Okeke et al., 2020) | 3 | + |
|  | **Ocupation** (Adekambi et al., 2020a; Jenkins et al., 2018; Mbanaso et al., 2011) | 3 | - |
|  | **Farming experience** (Acheampong et al., 2018; Ekwe, 2006; Kiiza et al., 2012; Kolawole et al., 2017**;** Mudombi, 2013**;** Mugumaarhahama et al., 2021**;** Okeke et al., 2020**;** Srinivas & Nedunchezhiyan, 2020) | 8 | +/- |
|  | **Annual income** (Agoh, 2021; Mudombi, 2013; Okeke et al., 2020) | 3 | + |
|  | **Farm income** (Ekwe, 2006; Mugumaarhahama et al., 2021; Okeke et al., 2020) | 3 | - |
|  | **Off-farm income** (Kaguongo et al., 2012; Kiiza et al., 2012; Mazuze, 2007; Mugumaarhahama et al., 2021) | 4 | - |
|  | **Livestock ownership** (Adekambi et al., 2020a; Mazuze, 2007) | 2 | + |
|  | **Farm size** (Acheampong et al., 2018**;** Adekambi et al., 2020a; Adekambi et al., 2020b**;** Ekwe, 2006**;** Josephine Namirimu et al., 2024**;** Kiiza et al., 2012**;** Kolawole et al., 2017**;** Mbanaso et al., 2011**;** Mazuze, 2007**;** Mugumaarhahama et al., 2021**;** Okeke et al., 2020) | 11 | + |
|  | **Land ownership (**Acheampong et al., 2018**;** Jogo et al., 2021**;** Kolawole et al., 2017**;** Mudombi, 2013**;** Mugumaarhahama et al., 2021**;** Srinivas & Nedunchezhiyan, 2020) | 6 | + |
|  | **Area under sweet potato** (Kaguongo et al., 2012**;** Mazuze, 2007**;** Srinivas & Nedunchezhiyan, 2020) | 3 | + |
|  | **Number of children/presence of children under 5 years** (Adekambi et al., 2020a**;** Jenkins et al., 2018**;** Jogo et al., 2021**;** Josephine Namirimu et al., 2024**;** Kaguongo et al., 2012**;** Mazuze, 2007) | 6 | + |
|  | **Presence of pregnant/lactating woman** (Adekambi et al., 2020b) | 1 | NA |
| **Institutional** | **Group membership** (Acheampong et al., 2018**;** Adekambi et al., 2020a; Adekambi et al., 2020b; Agoh, 2021**;** Jenkins et al., 2018**;** Kolawole et al., 2017**;** Mbanaso et al., 2011**;** Mugumaarhahama et al., 2021; Okeke et al., 2020) | 9 | + |
|  | **Extension service** (Acheampong et al., 2018**;** Adekambi et al., 2020a**;** Agoh, 2021**;** Kiiza et al., 2012**;** Kolawole et al., 2017**;** Mbanaso et al., 2011**;** Mazuze, 2007**;** Mudombi, 2013; Mugumaarhahama et al., 2021**;** Okeke et al., 2020) | 10 | + |
|  | **Training access (**Adekambi et al., 2020a; Adekambi et al., 2020b**;** Kaguongo et al., 2012**;** Kiiza et al., 2012**;** Mazuze, 2007**;** Mudombi, 2013**;** Mugumaarhahama et al., 2021) | 7 | + |
|  | **Access to credit (**Agoh, 2021**;** Mbanaso et al., 2011**;** Mugumaarhahama et al., 2021) | 3 | + |
|  | **Access of information** (Jenkins et al., 2018; Mudombi, 2013) | 2 | + |
|  | **Marketing distance** (Acheampong et al., 2018; Mugumaarhahama et al., 2021) | 2 | NA |
|  | **Distance to the nearest agricultural field office** (Adekambi et al., 2020a; Adekambi et al., 2020b) | 2 | - |
|  | **Distance to the nearest main road** (Adekambi et al., 2020a; Adekambi et al., 2020b) | 2 | NA |
|  | **Field to house distance** (Mugumaarhahama et al., 2021) | 1 | NA |
|  | **Holds a leadership position** (Jenkins et al., 2018; Mudombi, 2013) | 2 | NA |
|  | **Access to planting material** (Jenkins et al., 2018) | 1 | + |
|  | **Frequency of vines distribution** (Mazuze, 2007) | 1 | NA |
|  | **Participation in cooking demonstrations** (Adekambi et al., 2020b) | 1 | + |
|  | **Participation in program/project activities** (Adekambi et al., 2020b; Kaguongo et al., 2012) | 2 | + |
|  | **Participation in on-farm trials** (Mudombi, 2013) | 1 | + |
|  | **Irrigation use** (Mudombi, 2013) | 1 | + |
|  | **Input exchange** (Mudombi, 2013) | 1 | + |
|  | **Vine purchase** (Jogo et al., 2021) | 1 | + |
| **Agronomic and post-harvest** | **Dry matter** (Adekambi et al., 2020b; Jogo et al., 2021) | 2 | + |
|  | **Maturity** (Adekambi et al., 2020a; Adekambi et al., 2020b; Jenkins et al., 2018; Jogo et al., 2021; Mudombi, 2013) | 5 | + |
|  | **Yield** (Adekambi et al., 2020a; Adekambi et al., 2020b; Jogo et al., 2021; Mudombi, 2013; Srinivas & Nedunchezhiyan, 2020) | 5 | + |
|  | **Drought tolerance** (Adekambi et al., 2020b; Jenkins et al., 2018; Jogo et al., 2021; Mudombi, 2013) | 4 | + |
|  | **Pest and disease resistance** (Adekambi et al., 2020a; Adekambi et al., 2020b; Jenkins et al., 2018; Mudombi, 2013; Mugumaarhahama et al., 2021) | 5 | + |
|  | **Quantity produced** (Agoh, 2021; Ekwe, 2006) | 2 | + |
|  | **Experience with varieties** (Mazuze, 2007) | 1 | NA |
|  | **Easy to establish with scarce rain** (Adekambi et al., 2020b) | 1 | + |
|  | **Easy to conserve vines during the long dry period** (Adekambi et al., 2020b) | 1 | NA |
|  | **Variety growing** (Josephine Namirimu et al., 2024) | 1 | - |
|  | **Production method** (Josephine Namirimu et al., 2024) | 1 | - |
|  | **Crop establishment** (Mudombi, 2013) | 1 | NA |
|  | **Sweet potato storage** (Josephine Namirimu et al., 2024; Jenkins et al., 2018) | 2 | +/- |
|  | **Storage performance** (Mudombi, 2013) | 1 | + |
|  | **Easy to store in the ground** (Adekambi et al., 2020b) | 1 | - |
|  | **Multiplication and retention capacity** (Mudombi, 2013) | 1 | + |
|  | **Appropriateness to the farming system** (Mudombi, 2013) | 1 | NA |
|  | **Cropping system** (Mugumaarhahama et al., 2021) | 1 | NA |
|  | **Access to planting material** (Mugumaarhahama et al., 2021) | 1 | NA |
|  | **Soil fertility status** (Mugumaarhahama et al., 2021) | 1 | NA |
|  | **Type of production system** (Srinivas & Nedunchezhiyan, 2020) | 1 | + |
| **Psychological, environmental, and geographic** | **Knowledge Vita A** (Jogo et al., 2021; Kaguongo et al., 2012) | 2 | + |
|  | **Awareness of sweet potato** (Acheampong et al., 2018; Mazuze, 2007) | 2 | + |
|  | **Processing experience** (Agoh, 2021; Mazuze, 2007) | 2 | + |
|  | **Vine constrained** (Kaguongo et al., 2012) | 1 | - |
|  | **Cosmopolitaness** (Mudombi, 2013) | 1 | NA |
|  | **Environmental conditions** (Jenkins et al., 2018; Mazuze, 2007) | 1 | + |
|  | **Average annual precipitation** (Mazuze, 2007) | 1 | + |
|  | **Location of the farmer** (Kaguongo et al., 2012) | 1 | + |
| **Consumption and commercialization** | **Taste** (Adekambi et al., 2020b; Jogo et al., 2021) | 2 | +/- |
|  | **Very sugary/sweet** (Adekambi et al., 2020a; Adekambi et al., 2020b) | 2 | + |
|  | **Ease of cooking** (Adekambi et al., 2020a; Adekambi et al., 2020b) | 2 | - |
|  | **Sweet potato root consumption** (Mazuze, 2007) | 1 | NA |
|  | **Organoleptic qualities** (Jenkins et al., 2018) | 1 | + |
|  | **Output sold** (Josephine Namirimu et al., 2024; Mazuze, 2007) | 2 | NA |
|  | **Unstable markets** (Jenkins et al., 2018) | 1 | + |
|  | **Cost of processing** (Agoh, 2021) | 1 | NA |
|  | **Processing equipment** (Agoh, 2021) | 1 | + |
|  | **Value addition** (Kaguongo et al., 2012) | 1 | + |
|  | **Market price/Output price** (Srinivas & Nedunchezhiyan, 2020) | 1 | - |

Note: NA=Not applicable
